# Response to OK-432 sclerotherapy in the treatment of cervical lymphangioma with submucosal extension to the airway^☆^

**DOI:** 10.1016/j.bjorl.2016.04.016

**Published:** 2016-06-01

**Authors:** Michelle Manzini, Cláudia Schweiger, Denise Manica, Gabriel Kuhl

**Affiliations:** Hospital de Clínicas de Porto Alegre (HCPA), Porto Alegre, RS, Brazil

## Introduction

Lymphangioma is a localized lymphatic malformation, that occurs more frequently in the head and neck. Its etiology remains unknown; some authors believe it consists of a congenital malformation of the lymphatic vessels, while others believe that some acquired factors lead to lymphatic obstruction, lymph retention, lymphangiectasia and proliferation. Its incidence is 1 case for 6000–16,000 live births.^1^

It can occur at any age, but approximately 50% of cases are present at birth and 90% are diagnosed by 2 years of age. Both genders are equally affected. The most common affected sites are lips, tongue and neck. Infection, trauma and bleeding can trigger rapid growth of the lesion.

We report the case of a child diagnosed with submucosal microcystic lymphangioma with pharyngeal lesions that required tracheostomy due to upper airway obstruction. Treatment is described with OK-432 that allowed subsequent decannulation.

## Case report

**Figure 1:**
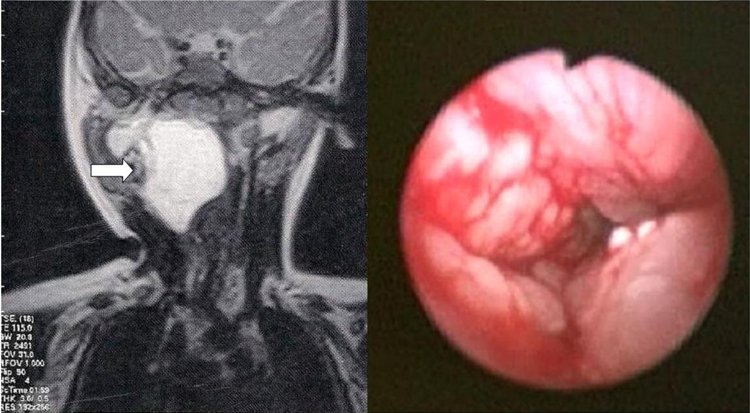
Magnetic resonance imaging, showing lesion in the retropharynx to the right (arrow). Endoscopic image of the upper airway (picture on the right).

A two-month old male patient was brought to the emergency department of a tertiary hospital in southern Brazil with stridor and respiratory effort. He was submitted to airway endoscopy under general anesthesia, which disclosed pharyngeal mucosa infiltration, from the nasopharynx to the hypopharynx, with upper airway obstruction (Fig. 1).

The cervical magnetic resonance imaging (MRI) showed a massive multiloculated lesion (4.9 × 4.3 × 3.6 cm^3^) located in the retropharyngeal space, on the right (Fig. 1 – arrow), extending posterior and anteriorly, involving the parapharyngeal space and the right parotid deep lobe, as well as anterior and laterally displacement of the contents of the carotid space, suggestive of lymphatic malformation (lymphangioma). This diagnosis was confirmed by pharyngeal mucosa biopsy.

The patient was tracheotomized to ensure airway patency. Sclerotherapy with OK-432 was chosen due to the impossibility of surgical resection. The cervical mass was treated by aspirating 5 mL, through cervical ultrasound, and injection of the same amount of picibanil at the site, resulting in its complete regression.

Two application cycles containing 2 mL of the medication were carried out in the airway mucosa, accounting for 4 sessions each cycle. He had fever up to 38 °C in the postoperative period, in addition to local pain and edema following some of the applications.

There was complete regression of the lesions after four applications and, thus, we chose to repeat the treatment cycle. After that, lesion improvement was observed and the patient was decannulated at three years of age. He remained asymptomatic after 18 months of follow-up following decannulation.

## Discussion

Lymphangioma is classified as macrocystic (more common in the neck), microcystic (more common in the tongue and cheek) and mixed. Spontaneous regression occurs in 1.6–16% of cases, with regression commonly being followed by recurrence.^2^

The diagnosis is made through clinical presentation and imaging tests, such as cervical ultrasound and MRI, which define the size and extent of the disease.^2^

The main goal of treatment is to restore function preservation and esthetic integrity.^3^ It should be individualized for each patient, depending on the size of the lesion, its location and the presence of complications – bleeding, recurrent infection, obstructive symptoms, esthetic deformities.^4^ Small lesions without functional or esthetic involvement require no treatment. Due to the fact that it is an infiltrative lesion without clear limits, the involvement of vital structures can complicate the management of this pathology and result in its incomplete resection, as well as nerve and blood vessel damage.^4^

In some mildly symptomatic cases there are reports of lesion involution in 15%–70% of cases; such children can be followed until the age of five years.^4^

Surgical resection is the definitive treatment and it is indicated in lesions larger than 3 cm, with progressive growth, bone erosion, dyspnea, dysphagia or esthetic deformity.^4^ The surgery is recommended after 6 months of age, unless the children develop compressive symptoms that may indicate an earlier surgery.^4^ Surgical treatment is also indicated for localized microcystic lesions.^2^ The lymphangioma can impair the airway by extrinsic compression or intrinsic involvement. Airway involvement is more common in childhood or after acute hemorrhage in a macrocystic lesion, requiring early treatment or tracheostomy. Prenatal detection can help plan the need for treatment outside the uterus, during delivery (Ex-Utero Intrapartum Treatment – EXIT).^3^

The surgical mortality rate can be up to 6%, and the rate of complications ranges from 19% to 33%. The recurrence rate is up to 53%.^4^

Other current treatments include radiation therapy, laser, and sclerotherapy with OK-432, doxycycline, sodium morrhuate, dextrose, tetracycline, hypertonic saline solution, acetic acid, ethanol, cyclophosphamide, interferon, fibrin glue, corticosteroids^4^ or boiling water.^1^

Radiotherapy may be indicated in recurrent and persistent cases that remain symptomatic or as an adjuvant to surgery. However, there is a risk of malignancy development, and a series of cases showed it was not an effective treatment.^2^

Endoscopic laser resection, either with CO_2_ or YAG, allows a more accurate lesion removal, without functional impairment.^2^ However, large lesions will possibly require other associated therapies. It could be an alternative treatment for palliative cases.^4^

The 95% or 100% alcohol solution is the most potent sclerosing agent, but can damage the vascular endothelium and denature the proteins and cause thrombosis. Adverse effects include nerve damage, pain, edema, systemic symptoms. The safe amount to be used in young children with large lesions is limited and often inefficient.^4^

Bleomycin is a cytotoxic antibiotic that induces degradation of deoxyribonucleic acid, causing lesion sclerosing and fibrosing.^2^ At low doses, symptoms can occur at the application site and a clinical picture similar to a common cold. The risk of using this drug is dose-dependent pulmonary fibrosis.^1^

Doxycycline is a broad-spectrum antibiotic that cause fibrosis and prevents lesion cell proliferation. Due to the discomfort of the injection, it should be applied under general anesthesia and pain-control medication are needed after the procedure.^3^ It seems to be more effective in the treatment of microcystic lymphangioma. It can cause tooth pigmentation.^4^

The injection of corticoids, such as triamcinolone,^2^ seems to be effective in intraoral cavernous hemangiomas, being effective in selected cases of cervical lymphangioma. Infection may be an adverse effect.

Few authors have reported on the use of fibrin glue, which would act as a hemostatic agent and would eliminate the dead spaces in the cysts; no side effects have been reported.^4^

Interferon-alpha 2a has shown to be effective in a small group of children, but it is not extensively used due to its adverse effects: fever, nausea, diarrhea, weight loss, liver enzyme abnormalities, alopecia, neutropenia, headaches.^4^

The intralesional use of cyclophosphamide was described in a study on life-threatening tumors. The most common adverse effects are: bone marrow suppression, hemorrhagic cystitis, liver dysfunction and long-term malignant transformation.^4^

OK-432 or picibanil is a biological preparation containing *Streptococcus pyogenes* strains treated with benzathine penicillin. After its injection into the lymphangioma, it stimulates the proliferation of lymphatic endothelial cells and obstruction of lymphatic channels. Its concentration is 0.1 mg/10 mL. The dose should not exceed 20 mL^2^ per application, although there have been reports on 30 mL use.^5^ It is believed that endothelial damage caused by picibanil is secondary to the activation of the host immune system. The biggest disadvantage of using it is the risk of hemodynamic repercussions, especially in those allergic to penicillin. Fever up to 39 °C often occurs within 6 h after injection, which remits in approximately 4 days with the use of antipyretic drugs,^1^ which was confirmed in the outcome of our patient.

OK-432 leads to complete response in macrocystic lesions, with a lower degree of response in mixed or microcystic lesions.^3^ Polycystic lesions show inferior results.^3^ Similarly, the suprahyoid macrocystic or microcystic lesions and infrahyoid microcystic lesions are more difficult to handle than infrahyoid macrocystic and posterior cervical lesions. The reason for this discrepancy is yet to be clarified.^3^

If the lesion does regress within 90 days, a new application with up to 20 mL can be performed.

Several reports have concluded that OK-432 is safe and effective for the treatment of lymphangiomas^5^ and it seems to reduce the need for adjuvant therapies.^3^

## Final considerations

There are few reports about the effects of treatment with OK-432 in microcystic submucosal lymphangiomas in patients who required tracheostomy. There is little evidence on each therapeutic measure and most of them are based on case reports or series of cases. This is the first report on the use of this medication in this type of lesion that allowed patient decannulation.

## Conflicts of interest

The authors declare no conflicts of interest.
